# Maternal determinants of optimal breastfeeding and complementary feeding and their association with child undernutrition in Malawi (2015–2016)

**DOI:** 10.1186/s12889-019-7877-8

**Published:** 2019-11-11

**Authors:** Christine N. Walters, Hasina Rakotomanana, Joel J. Komakech, Barbara J. Stoecker

**Affiliations:** Department of Nutritional Sciences, Oklahoma State University, Stillwater, OK USA

**Keywords:** IYCF, Child undernutrition, Maternal determinants, Malawi

## Abstract

**Background:**

Optimal breastfeeding and complementary feeding practices are critical to prevent child undernutrition. Despite the occurrence of child undernutrition and widespread suboptimal feeding practices in Malawi, the association of breastfeeding and complementary feeding practices and undernutrition among Malawian children remains unclear. The purpose of the study was to determine the current breastfeeding and complementary feeding practices, to identify maternal determinants of each practice, and to analyze the associations between breastfeeding and complementary feeding practices with stunting, underweight, and wasting.

**Methods:**

The most recent Malawi Demographic Health Survey (2015–2016) was used and data for 2294 children aged 0–23 months were included. A conceptual framework of five maternal domains: sociodemographic, health status, health behaviors, women’s empowerment, and media exposure was used. Each domain contained exposure variables and the WHO Infant and Young Child Feeding indicators were used as outcome variables. All analyses were adjusted for clustering, and variables that reached a *p*-value of < 0.05 were considered significant in the final model.

**Results:**

Among children, 30.8% were stunted, 9.9% were underweight, and 3.7% were wasted. Many (78%) were breastfed within the first hour of birth, 89% were breastfed until their first birthday, yet 40% were not exclusively breastfed to 6 months. Only 32% met minimum dietary diversity, 23% met minimum meal frequency, 12% met minimum acceptable diet and 12% consumed iron-rich foods. Children whose mothers lived in urban areas were less likely to be breastfed within 1 hour of birth but more likely to meet minimum dietary diversity. Children whose mothers listened to radio were more likely to meet minimum meal frequency. Children (13–23 months) who met minimum meal frequency and minimum acceptable diet were less likely to be underweight.

**Conclusions:**

Optimal breastfeeding and complementary feeding practices in Malawi remain suboptimal and child undernutrition remains problematic. Maternal characteristics from the five domains were significantly associated with optimal breastfeeding and complementary feeding indicators. Knowledge of these maternal determinants can assist in improving nutrition policies and interventions that aim to impact breastfeeding and complementary feeding practices and child growth in Malawi.

## Background

Worldwide, undernutrition is associated with nearly 45% of child mortality [1]. Undernutrition during the first 2 years of life is a determinant of childhood stunting and non-communicable diseases in adulthood [2]. Inadequate nutrition during the first 1000 days hinders physical and cognitive development and increases the risk of child mortality [1]. Consequently, adequate nutrition during this critical period is vital for healthy growth and development [2]. Optimal breastfeeding and complementary feeding practices are necessary for the prevention of child undernutrition [3]. The World Health Organization (WHO) recommends exclusive breastfeeding for the first 6 months of life with continued breastfeeding for 2 years or beyond and timely introduction of safe, appropriate, and nutritionally adequate complementary foods [4]. Suboptimal feeding practices are associated with the prevalence of undernutrition as well as increased morbidity and mortality among children in low-income countries [5]. In efforts to improve feeding practices, the WHO established Infant and Young Child Feeding (IYCF) indicators to measure adherence to recommendations at the population level [3].

In Malawi, the World Food Programme reported in 2015 that 23% of child deaths were directly associated with undernutrition [6]. In 2010, 47% of children between 0 and 59 months were stunted, 13% underweight, and 4% wasted [7]. The Malawian Ministry of Health and Population developed Infant and Young Child Nutrition Policy and Guidelines in response to high child malnutrition and mortality rates [8]. Despite limited improvements since the introduction of the policy, suboptimal complementary feeding practices remain a concern in Malawi [7]. In the latest DHS survey, only 29% of 6–23 month old infants and children achieved the minimum dietary diversity (MDD) and 19% achieved the minimum acceptable diet (MAD) [7]. Findings from the Chikwawa District in Malawi showed that 65% of infants received complementary foods by 3 months, much earlier than the WHO recommendations [9]. Another study found that 35% of Malawian mothers did not initiate breastfeeding within the first hour of birth and only 7.5% exclusively breastfed for the first 6 months [10].

Due to suboptimal adherence to the WHO feeding recommendations and the occurrence of child undernutrition in Malawi, understanding the maternal determinants associated with optimal breastfeeding and complementary feeding practices is essential for developing effective nutrition interventions and improving nutrition policies. Studies conducted outside of Malawi have found that maternal education, literacy, and wealth status have been associated with breastfeeding and complementary feeding practices [11–13]. Furthermore, prior evidence from sub-Saharan African countries shows that empowered women were more likely to follow complementary feeding recommendations [14, 15]. Additionally, mothers’ exposure to mass media, such as television, radio, or newspaper was a determinant of optimal feeding practices in Burkina Faso [16], Tanzania [17], and Madagascar [12].

Evidence suggests associations between the IYCF indicators and child growth vary by indicator [18] and by country [19]. Additionally, results remain inconsistent on the associations between child undernutrition and feeding practices in Malawi. In a cross-sectional study, exclusively breastfed infants (0–6 months) had significantly higher length-for-age (LAZ) and weight-for-age (WAZ) compared to those who were not exclusively breastfed [20]. Similarly, in another cross-sectional study, duration of exclusive breastfeeding was positively associated with LAZ among infants 6–8 months but not among infants less than 6 months or with WAZ or weight-for-height z-score (WHZ) in infants 0–12 months [10]. Therefore, analyzing the association between IYCF and child growth using nationally representative data should provide a broader understanding of the discrepancies found across Malawian studies. Despite high rates of child undernutrition, the maternal determinants of optimal breastfeeding and complementary feeding practices and their association with child undernutrition in Malawi remain unclear. Studies have not investigated all the maternal characteristics from the five proposed domains as determinants of optimal breastfeeding and complementary feeding practices in Malawi. Therefore, this study has three main objectives: 1) to determine the situation of breastfeeding and complementary feeding practices in Malawi, 2) identify the maternal determinants of each IYCF indicator, and 3) to analyze the association between each IYCF indicator and stunting, underweight, and wasting. To our knowledge, this study is the first to use national data to investigate maternal determinants of both optimal breastfeeding and complementary feeding practices and their association with underweight, stunting, and wasting among Malawian children. The results provide a basis for evidence-based recommendations to inform policies and interventions that aim to improve breastfeeding and complementary feeding practices and child nutrition in Malawi.

## Methods

**Fig. 1:**
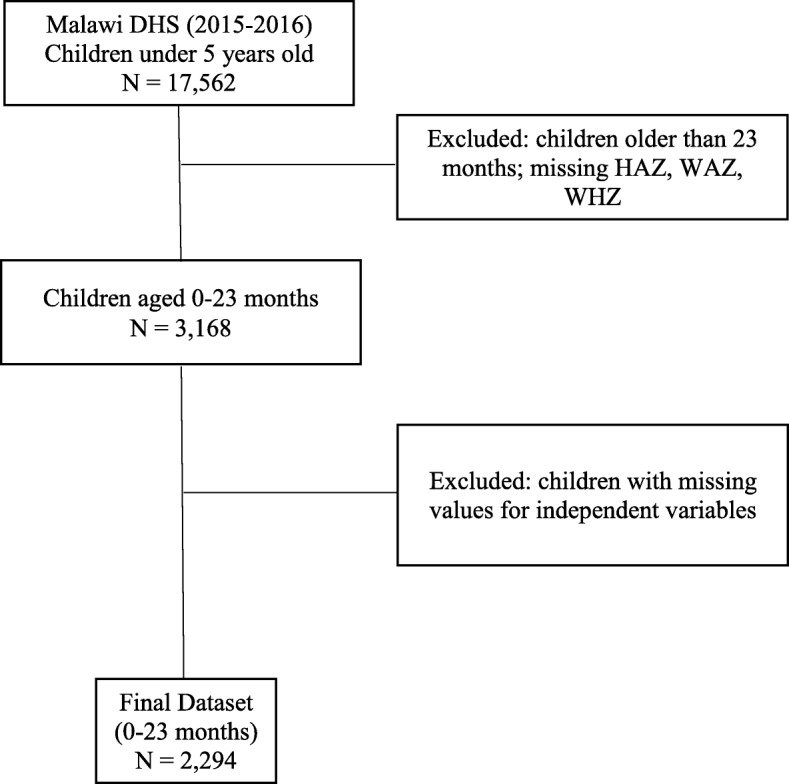
Sampling method detailing inclusion of children

**Fig. 2:**
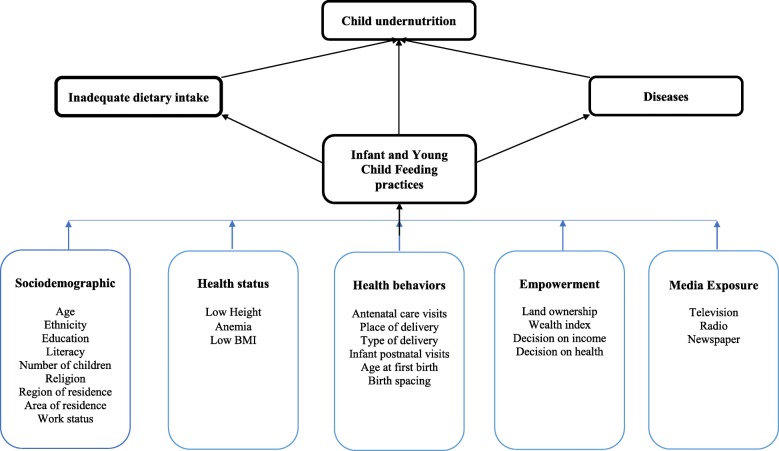
Framework on the influence of maternal characteristics from five domains on infant and young child feeding practices and child undernutrition (adapted from Black et al., 2008 and Ickes et al., 2015)

The Malawi Demographic and Health Survey (2015–2016) collected nationally representative data about maternal and child health issues affecting the Malawian population. The Malawi Population and Housing Census sampling frame was used. The two-stage stratified sampling method allows estimations for the country, region, district, and urban and rural areas. A total of 27,516 households were selected. Of these households, 26,361 were interviewed (99% response rate). A comprehensive overview of the sampling design and methodology are described in the MDHS 2015–2016 Report [21]. For the purpose of this study, the child dataset was used, and the sample included 2294 children aged 0–23 months (Fig. 1). A conceptual framework (Fig. 2) was developed based on the work of Black et al. [5] and Ickes et al. [11] to evaluate the influence of maternal characteristics on feeding practices. Maternal characteristics were categorized into five key domains: sociodemographic, health status, health behaviors, women’s empowerment, and media exposure. Each domain contains exposure variables that have been associated with optimal feeding practices in other countries [6, 7, 13–15, 17–19]. The WHO Infant and Young Child Feeding indicators were used as outcomes.

The WHO growth standards were used to determine weight-for-age, length-for-age, and weight-for-length z-scores [22]. Binary variables based on the IYCF core indicators [3] were created to measure seven outcomes: early initiation of breastfeeding, exclusive breastfeeding under 6 months, continued breastfeeding at 1 year, minimum dietary diversity, minimum meal frequency, minimum acceptable diet, and consumption of iron-rich or iron-fortified foods.

Child and maternal characteristics are presented using descriptive statistics and weighted frequencies. Bivariate logistic regressions were performed on each maternal variable to determine their association with each IYCF indicator. Multivariate logistic regression analyses were then conducted for each domain of maternal characteristics with all the variables that reached a *p*-value < 0.10 in the bivariate analyses. The final multivariate model included all variables that reached a p-value of < 0.05 in the multivariate analyses for each domain. Logistic regressions adjusted for clustering were used to analyze the associations between each IYCF indicator and wasting, stunting, and underweight. Using the Variance Inflation Factor (VIF) tolerance test, multicollinearity was tested among explanatory variables in each model and all results were within normal limits. SAS, v. 9.4, was used for all analyses.

## Results

### Child health and nutritional status

Among child participants, 30.8% were stunted, 9.9% were underweight, and 3.7% were wasted (Table 1). Most children (80%) were anemic and 49.5% had moderate anemia. Morbidity was common; 31.1% of children had diarrhea and 34.5% had a fever in the 2 weeks prior to the survey.

**Table 1:** Child and maternal characteristics, Malawi 2015–2016

Variables	Percentage (%)^a^
*Child characteristics*	
Nutritional status	
Stunted (*n* = 2222)	30.8
Wasted (*n* = 2221)	3.7
Underweight (*n* = 2224)	9.9
Anemic (*n* = 1670)	80.0
Severe (Hb < 7.0 g/dL)	3.7
Moderate (Hb 7.0–9.9 g/dL)	49.5
Mild (Hb 10.0–11.0 g/dL)	26.8
Morbidity (*n* = 2281)	
Diarrhea	31.1
Fever	34.5
Cough	27.3
*Maternal characteristics*	
Maternal education (*n* = 2281)	
No education	11.2
Some primary	65.9
Secondary or higher	22.9
Maternal literacy (*n* = 2281)	
Cannot read	30.8
Able to read parts of sentence	9.8
Able to read whole sentence	59.4
Maternal occupation (*n* = 2281)	
Not working outside the home	36.9
Working outside the home	63.1
Marital status (*n* = 2281)	
Unmarried	21.3
Married	78.7
Ethnicity (*n* = 2222)	
Chewa	34.6
Tumbuka	8.4
Lomwe	18.0
Yao	15.8
Ngoni	11.7
Other	11.5
Religion (*n* = 2279)	
Christian	83.8
Muslim	15.6
Other	0.6
Exposure to media (*n* = 2222)	
Television	
Not at all	82.9
Rarely (Less than once a week)	9.2
At least once a week	7.9
*Maternal characteristics (continued)* (*n* = 2281)	
Radio	
Not at all	53.2
Rarely (Less than once a week)	19.3
At least once a week	27.5
Newspaper	
Not at all	83.9
Rarely (Less than once a week)	10.4
At least once a week	5.7
Wealth index	
Poorest	25.0
Poorer	22.7
Middle	19.9
Richer	15.8
Richest	16.6
Area of residence	
Urban	14.8
Rural	85.2
Region of residence	
Northern	11.2
Central	42.0
Southern	46.8

^a^Weighted frequencies

### Maternal characteristics

Maternal characteristics of participants are presented in Table 1. Completion of some primary school was the highest level of education for 65.9% of the mothers and many (30.8%) could not read. More than half (63.1%) of mothers were working outside the home and 78.7% were married. Most (85.2%) were living in rural areas and 47.7% were in the two lowest wealth quintiles. Mean (SD) maternal age was 26.5 (6.7) years and mean age at first birth was 18.7 (2.8) years (data not shown).

### Breastfeeding and complementary child feeding practices

**Fig. 3:**
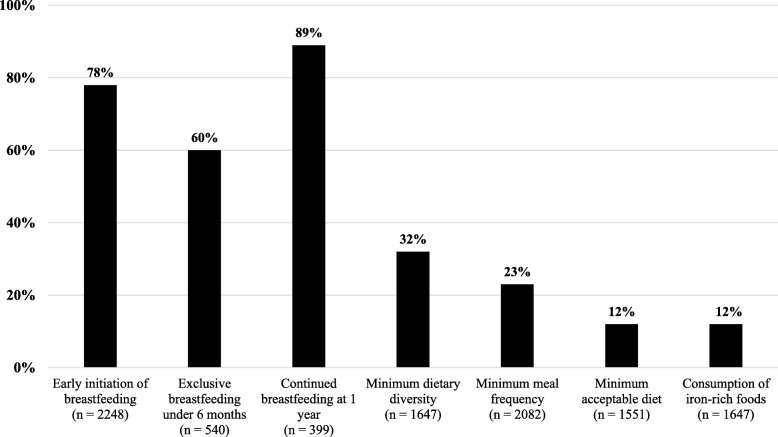
Optimal breastfeeding and complementary feeding practices in Malawi (2015–2016)

The majority (78%) of women breastfed within the first hour after birth and 89% continued breastfeeding until the child’s first birthday (Fig. 3). However, 40% did not exclusively breastfeed for 6 months. Only 32% of the children 6–23 months of age ate foods from four or more food groups and only 23% met minimum meal frequency (MMF). Very few (12%) met minimum acceptable diet (MAD) or consumed iron-rich or iron-fortified foods.

### Maternal determinants of breastfeeding and complementary feeding practices

#### Sociodemographic determinants

Table 2 presents the significant determinants of breastfeeding and complementary feeding practices in the final adjusted model. For the sociodemographic domain, the significant determinants were ethnicity, number of children, region, and area of residence. Children whose mothers identified as Ngoni were less likely to meet MAD [AOR = 0.58 (0.33–0.99), *p* < 0.05], but a higher number of children was associated with higher likelihood of meeting MAD [AOR = 1.12 (1.02–1.23), *p* < 0.05]. Mothers living in the central region [AOR = 0.64 (95% CI:0.43–0.95), *p* < 0.05] and urban areas [AOR = 0.46 (0.29–0.72), *p* < 0.001] were less likely to initiate breastfeeding within 1 hour of birth. Children of mothers in urban areas were more likely to meet minimum dietary diversity (MDD) [AOR = 13.50 (4.25–42.89), *p* < 0.0001] and those in the Southern region were more likely to have MMF [AOR = 1.74 (1.04–2.92), *p* < 0.05].

**Table 2:** Maternal determinants of optimal breastfeeding and complementary feeding practices, Malawi 2015–2016

Maternal Characteristic	Early Initiation of Breastfeeding	Exclusive Breastfeeding Under 6 Months	Continued Breastfeeding at 1 Year	Minimum Dietary Diversity	Minimum Meal Frequency	Minimum Acceptable Diet	Iron-Rich Foods
Sociodemographic							
Ethnicity							
Chewa				1		1	
Tumbuka				0.97 (0.31–3.08)		1.39 (0.79–2.46)	
Lomwe				1.20 (0.27–5.30)		0.66 (0.38–1.15)	
Yao				0.98 (0.29–3.32)		0.65 (0.36–1.16)	
Ngoni				0.86 (0.25–2.97)		0.58 (0.33–0.99)*	
Other				2.40 (0.49–12.03)		0.61 (0.32–1.15)	
Number of children						1.12 (1.02–1.23)*	
Region							
Northern	1				1		
Central	0.64 (0.43–0.95)*				0.95 (0.55–1.65)		
Southern	1.12 (0.76–1.67)				1.74 (1.04–2.92)*		
Area of residence							
Rural	1			1			1
Urban	0.46 (0.29–0.72)***			13.50 (4.25–42.89)****			1.08 (0.52–2.26)
Health status							
Maternal height							
Height ≤ 150 cm	1						
Height > 150 cm	1.51 (1.06–2.15)*						
Maternal BMI							
BMI < 18.5 kg/m^2^					1		
BMI ≥ 18.5 kg/m^2^					2.76 (1.02–7.46)*		
Health behaviors							
Place of delivery							
Home				1			
Government				0.72 (0.18–2.93)			
Private				1.28 (0.20–8.10)			
Delivery							
Vaginal delivery	1						
Caesarean delivery	0.32 (0.20–0.51)****						
Infant postnatal checks							
No				1		1	
Yes				1.55 (0.61–3.89)		0.59 (0.41–0.85)*	
Empowerment							
Landownership							
Does not own	1			1			
Owns	1.02 (0.74–1.41)			1.64 (0.49–5.44)			
Wealth index							
Poorest	1			1			1
Poorer	0.95 (0.64–1.43)			0.77 (0.17–3.43)			1.27 (0.59–2.69)
Middle	0.94 (0.63–1.41)			0.66 (0.18–2.42)			1.49 (0.75–2.96)
Richer	1.16 (0.72–1.86)			0.49 (0.12–2.25)			1.82 (0.85–3.89)
Richest	0.97 (0.59–1.60)			2.12 (0.40–11.23)			5.11 (2.12–12.33)***
Decision on income							
Respondent alone					1		
Respondent and husband/partner					0.68 (0.40–1.13)		
Husband/partner alone					0.59 (0.35–1.01)		
Decision on health							
Respondent alone						1	
Respondent and husband/partner						1.25 (0.79–1.97)	
Husband/partner alone						1.48 (0.93–2.37)	
Media Exposure							
TV							
Not at all				1			1
Rarely (Less than once a week)				0.62 (0.17–2.25)			0.72 (0.33–1.59)
At least once a week				0.35 (0.09–1.29)			1.28 (0.54–3.07)
Radio							
Not at all					1	1	
Rarely (Less than once a week)					2.59 (1.65–4.07)****	0.52 (0.36–0.80)*	
At least once a week					2.56 (1.57–4.23)***	0.48 (0.29–0.77)*	

Final adjusted model: results expressed as adjusted odds ratio AOR (95% Confidence Interval); *p-value < 0.05, **p-value< 0.01, ***p-value< 0.001, ****p-value< 0.0001

#### Health status determinants

Maternal height and body mass index (BMI) status were significant determinants of early initiation of breastfeeding and minimum meal frequency, respectively (Table 2). Maternal height taller than 150 cm was associated with a higher likelihood of initiating breastfeeding within the first hour of birth [AOR = 1.51 (1.06–2.15), *p* < 0.05]. Mothers with a BMI ≥ 18.5 kg/m^2^ had nearly three times the likelihood of their children meeting minimum meal frequency [AOR = 2.76 (1.02–7.46), *p* < 0.05). Maternal anemia was not associated with any of the IYCF indicators.

#### Health behaviors determinants

In the health behaviors domain, type of delivery and infant postnatal checks were significant determinants (Table 2). Mothers who had a Caesarean delivery were less likely to initiate breastfeeding immediately after birth [AOR = 0.32 (0.20–0.51), *p* < 0.0001]. Additionally, children of mothers who had postnatal checks were less likely to meet MAD [AOR = 0.59 (0.41–0.85), *p* < 0.05].

#### Women’s empowerment determinants

Table 2 shows the significant women’s empowerment determinants for optimal IYCF practices and only wealth remained significant in the final models. Children of mothers who were in the highest wealth index were more likely to consume iron-rich foods [AOR = 5.11 (2.12–12.33), *p* < 0.001].

#### Media exposure determinants

Exposure to radio was the only significant media exposure determinant in the final models and was associated with both MMF and MAD (Table 2). Children whose mothers listened to radio rarely [AOR = 2.59 (1.65–4.07), *p* < 0.0001] and those who listened at least once a week [AOR = 2.56 (1.57–4.23), *p* < 0.001] were more than two times more likely to meet MMF compared to children whose mothers never listened to radio. However, children whose mothers listened to radio rarely [AOR = 0.52 (0.36–0.80), *p* < 0.05] and those who listened at least once a week [AOR = 0.48 (0.29–0.77), *p* < 0.05] were less than half as likely to meet MAD compared to children whose mothers never listened to radio.

### Breastfeeding and complementary feeding practices and child nutritional status

Children (13–23 months) who met MMF or MAD were less likely to be underweight [AOR = 0.41 (0.21–0.79), *p* < 0.01)] and [AOR = 0.23 (0.08–0.65), *p* < 0.01] (Table 3). The IYCF indicators were not associated with stunting or wasting.

**Table 3:** Breastfeeding and complementary feeding practices and their association with stunting, underweight, and wasting in Malawian children, 2015–2016

IYCF Indicators	Age	Stunting	Underweight	Wasting
Early initiation of breastfeeding^a^	0–23	0.84 (0.63–1.13)	1.13 (0.73–1.74)	1.05 (0.55–1.98)
Exclusive breastfeeding under six months^b^	0–5	1.63 (0.91–2.93)	1.22 (0.47–3.18)	0.37 (0.11–1.41)
Continued breastfeeding at one year^c^	12–15	1.95 (0.65–5.87)	0.71 (0.20–2.53)	0.69 (0.08–6.22)
Minimum dietary diversity^d^	6–12	0.99 (0.56–1.75)	1.78 (0.86–3.67)	0.96 (0.53–1.74)
	13–23	0.73 (0.49–1.09)	1.08 (0.61–1.88)	1.15 (0.46–2.86)
Minimum meal frequency^e^	6–12	1.00 (0.63–1.59)	1.25 (0.67–2.32)	0.94 (0.44–2.05)
	13–23	0.86 (0.57–1.30)	0.41 (0.21–0.79)*	1.54 (0.63–3.86)
Minimum acceptable diet^f^	6–12	1.06 (0.52–2.16)	1.92 (0.72–5.13)	0.62 (0.16–2.45)
	13–23	0.62 (0.31–1.24)	0.23 (0.08–0.65)*	0.91 (0.24–3.41)
Consumption of iron-rich foods^g^	6–12	0.89 (0.34–2.33)	0.98 (0.17–5.54)	1.11 (0.31–4.01)
	13–23	1.26 (0.78–2.05)	0.82 (0.40–1.67)	0.51 (0.12–2.24)

Results expressed as adjusted odds ratio AOR (95% Confidence Interval); *p-value < 0.01

Adjusted for clustering, child age, child sex, wealth index, and maternal education

^a^Children born in the last 24 months who were breastfed within 1 hour of birth

^b^Infants 0–5 months of age who were fed exclusively with breast milk (no formula or complementary foods) the previous day

^c^Children 12–15 months of age who were fed breastmilk the previous day

^d^Children 6–23 months of age who received foods from 4 or more food groups the previous day

^e^Breastfed and non-breastfed children 6–23 months of age who received solid, semi-solid, or soft foods (but also including milk feeds for non-breastfed children) the minimum number of times or more the previous day (Minimum is defined as: 2 times for breastfed infants (6–8 months), 3 times for breastfed children (9–23 months), 4 times for non-breastfed children (6–23 months)

^f^Breastfed children 6–23 months of age who had at least the minimum dietary diversity and the minimum meal frequency during the previous day and non-breastfed children 6–23 months of age who received at least 2 milk feedings and had at least the minimum dietary diversity not including milk feeds and the minimum meal frequency

^g^Children 6–23 months of age who receive an iron-rich food or iron-fortified food that is specially designed for infants and young children, or that is fortified in the home the previous day

## Discussion

### Situation of breastfeeding and complementary feeding practices

Despite adherence to early initiation of breastfeeding (78%) and continued breastfeeding by the child’s first birthday (89%), 40% of Malawian mothers were not exclusively breastfeeding for the first 6 months of their infant’s life. Previous research in Malawi has shown that even though most (81%) mothers reported their breastfeeding beliefs were influenced by healthcare workers, only 40% of mothers believed that infants should be exclusively breastfed for the first 6 months [23]. Another study in Malawi, found it was common for Malawian infants to be introduced to water, porridge, and herbal infusions before 6 months of age [24]. Furthermore, evidenced has shown that some Malawian fathers reportedly added formula to the infant’s diet [25]. A recent study discussed how Malawian health workers may provide insufficient advice or that mothers and fathers misinterpreted breastfeeding recommendations [25]. Therefore, it is important to help Malawian women find ways to overcome the barriers they face to exclusively breastfeeding for the first 6 months of their child’s life. These results also indicate a strong need for more focus on intensive training for health workers on the benefits of exclusive breastfeeding and the overall promotion of exclusive breastfeeding in Malawi.

The percentage of Malawian children meeting optimal complementary feeding practices including MDD, MMF, MAD, and consumption of iron-rich foods remains low. According to a report in 2016 from the Malawi Vulnerability Assessment Committee, maize production has decreased dramatically since 2010 and the number of people not able to meet their annual minimum food requirements has increased [26]. In Malawi, household food insecurity has been associated with less diverse diets [27]; thus, household food insecurity may have been a factor in the decreased adherence to MMF, MAD, and consumption of iron-rich foods.

### Maternal determinants of optimal breastfeeding and complementary feeding practices

#### Sociodemographic determinants

Mothers living in urban areas were less likely to initiate breastfeeding within 1 hour of birth than mothers from urban areas. Health facilities in urban areas should be encouraging early initiation of breastfeeding, especially since the Baby Friendly Hospital Initiative has been adopted by some health facilities throughout Malawi [28]. Malawian women delivering in facilities should receive breastfeeding education and support from trained healthcare staff.

Children from urban areas were more likely to meet MDD compared to children living in rural areas. Previous studies found diversity of diets was higher in urban compared to rural areas and food insecurity was less common in urban areas [29]. Malawians in urban areas may have greater access and ability to consume a more diverse diet. Overall, it appears that breastfeeding interventions are more necessary in urban areas whereas complementary feeding interventions are needed in rural areas.

Children whose mothers identified as Ngoni were less likely to meet MAD. Community characteristics, including ethnicity and lineage, are important factors that may influence child nutrition in Malawi [29]. Due to the number of ethnic groups in Malawi, it may be critical to consider their unique characteristics when designing nutrition interventions and policies aimed at improving breastfeeding and complementary feeding practices.

#### Health status determinants

Maternal height (>150 cm) was associated with a higher likelihood of initiating breastfeeding within the first hour of birth. One possibility is that mothers with short stature are at a higher risk for having a Caesarean section [30]. Therefore, the association between maternal height and early initiation of breastfeeding may be at least partially because mothers with taller stature are less likely to have a Caesarean section and thus, more likely to initiate breastfeeding within the first hour of birth.

Furthermore, a maternal BMI ≥ 18.5 was associated with more than double the likelihood of their children meeting minimum meal frequency. Although not all studies have found significance between maternal BMI and child feeding [17], maternal nutritional status has been linked with improved child nutritional status [31]. Therefore, it is plausible that women with a healthy BMI may have the health knowledge, health behaviors, or financial ability to access enough food to feed their child. Overall, ensuring that women have better nutritional status, even in the pre-conception phase and throughout the duration of pregnancy, appears to be an important factor to address when promoting early initiation of breastfeeding and MMF in Malawi.

#### Health behavior determinants

Mothers who had a Caesarean delivery were less likely to initiate breastfeeding immediately after birth. Similar results in a systematic review and meta-analysis revealed that rates of breastfeeding within 1 hour of birth after Caesarean deliveries were significantly lower compared to vaginal deliveries [32]. Likewise, delayed onset of lactation was significantly higher in mothers who had a Caesarean delivery compared to vaginal delivery [33]. The WHO only recommends a Caesarean section when it is medically justified, which would also seem to support better IYCF practices [34]. Surprisingly, infant postnatal checks were negatively associated with minimum acceptable diet. This would appear to contradict other studies reporting a lack of postnatal visits was associated with suboptimal complementary feeding practices [17]. However, according to an assessment of postnatal care services in Malawi, a group education approach was utilized, and mothers’ needs were not met [35]. Because women should attend postnatal care, these results indicate there may be a need to improve training of healthcare workers for optimal strategies to reach mothers and improve postnatal care policies to increase the quality of the care being provide.

#### Women’s empowerment determinants

Wealth was the only maternal determinant from the women’s empowerment domain that remained significant in the final models. Children of mothers who were in the highest wealth category were more likely to consume iron-rich foods, which is consistent with previous results from Madagascar [12]. Households with more wealth likely have the ability to access, purchase, and consume more expensive foods, including iron-rich foods.

#### Media exposure determinants

Mother’s exposure to radio was associated with a significantly higher likelihood of children meeting MMF but a lower likelihood of MAD. These conflicting results indicate a need for further research in understanding how media exposure can influence complementary feeding practices. Previous studies have found that children of Ethiopian mothers who received IYCF information via mass media had higher diet quality [37] and Malagasy children whose mothers had greater media exposure had lower odds of inadequate dietary diversity [12]. Exposure to media such as radio, may be a viable mechanism for delivering nutrition education to mothers in low-income countries. In Malawi, organizations have already utilized media as a channel for spreading nutrition awareness and knowledge [38]. Malawian women exposed to a community-led mass media campaign were more likely to utilize maternal health services, including antenatal and postnatal care [39]. While it appears media may be important in improving child feeding practices, it is also necessary to find ways to increase access and availability of media to Malawian mothers because newspaper, radio, and television remain unavailable to more than half of Malawian women.

### Breastfeeding and complementary feeding practices and child nutritional status

Our findings add to the evidence that diet quality and frequency of complementary feeding are essential for higher weight-for-age z-scores. Strategies to help children meet minimum acceptable diet and minimum meal frequency are likely to assist in reducing the prevalence of underweight among young Malawian children. The IYCF indicators were not associated with stunting or wasting; however, it has been acknowledged that some of the indicators are lacking in sensitivity and specificity [19].

### Implications and future research

Because exclusive breastfeeding remains suboptimal in Malawi, future research should consider in-depth qualitative assessments of the breastfeeding barriers for Malawian women. A concerning finding was that attending postnatal care visits decreased the likelihood of children meeting MAD. Previous studies in Malawi have reported potential gaps in postnatal care and highlight the need to ensure that nutrition education provided by health care workers is being adequately understood [25, 27]. Postnatal care in Malawian health care facilities should be evaluated and effective communication mechanisms for nutrition education must be identified. Due to the inconsistent findings regarding media exposure, future research should explore the influence of media on breastfeeding and complementary feeding practices and consider how to increase the access and availability of mass media as well as exploring what technologies and approaches may be most effective at communicating information to Malawian caregivers.

The lower likelihood of being underweight that was associated with meeting MMF or MAD, suggests that nutrition interventions and policies that focus on promoting diet quality and frequency are important for optimal weight gain. Despite stunting remaining a concern among Malawian children, our findings showed no significant associations between breastfeeding and complementary feeding practices and child stunting. Given the high stunting rates in Malawi, a thorough examination of the multiple possible determinants of stunting is critical in order to effectively develop policies, interventions, and programs to address the problem.

Despite the use of nationally representative data, the study had some limitations. The data are cross-sectional and therefore, causal relationships are not exposed. The use of a 24-h recall to assess feeding practices may not reflect daily or seasonal diet variability. Due to the data collection techniques utilized, there is a possibility of recall bias, self-reporting errors, and social desirability bias. Data regarding maternal IYCF knowledge was not collected and is likely an important factor influencing feeding practices.

## Conclusions

Overall, breastfeeding and complementary feeding practices in Malawi remain suboptimal while undernutrition remains a health concern among Malawian children. The findings from this study show that Malawian children (aged 13–23 months) meeting MMF or MAD recommendations were less likely to be underweight. Maternal characteristics from each of the five domains were significantly associated with one or more breastfeeding or complementary feeding practice. These variables included ethnicity, number of children, region and area of residence, maternal height, maternal BMI, type of delivery, postnatal checks, wealth index and radio use. Knowledge of these maternal determinants can assist in improving nutrition policies and interventions that aim to improve breastfeeding and complementary feeding practices and child growth in Malawi.

## Data Availability

The data analyzed are available upon request and approval from the Demographic and Health Survey (DHS) at https://www.dhsprogram.com/data/dataset/Malawi_Standard-DHS_2015.cfm?flag=0.

## Abbreviations

AOR: Adjusted odds ratio
BMI: Body mass index
CI: Confidence interval
DHS: Demographic and Health Survey
IYCF: Infant and Young Child Feeding
LAZ: Length-for-age z-score
MAD: Minimum acceptable diet
MDD: Minimum dietary diversity
MMF: Minimum meal frequency
SAS: Statistical Analysis System
WAZ: Weight-for-age z-score
WHO: World Health Organization
WHZ: Weight-for-height z-score
